# Design and evaluation of a comprehensive training program for hospital-based clinical pharmacists - various, active, and work-integrated learning

**DOI:** 10.1016/j.rcsop.2025.100677

**Published:** 2025-10-27

**Authors:** Marianne Lea, Elin Trapnes, Hanne Steen, Nina Bjerketveit Ødegaard

**Affiliations:** a Department of Clinical Pharmacy and Counselling, Oslo Hospital Pharmacy, Hospital Pharmacies Enterprise, South Eastern Norway, Oslo, Norway; b Department of Pharmacy, Section for Pharmacology and Pharmaceutical Biosciences, University of Oslo, Oslo, Norway; c BI Norwegian Business School, Oslo, Norway

**Keywords:** Education, Pharmacy, Continuing, Preceptorship, Mentoring, Medication reconciliation, Medication review

## Abstract

**Background:**

Medication reconciliation and -reviews are not emphasized in the pharmacy curriculums in Norway and graduated pharmacists are at novice level. Therefore, a postgraduate training program for hospital-based clinical pharmacists was developed. The aim of this paper is to describe the program design and evaluation of the last four years (2021–2024).

**Method:**

The training program, established in 2012, used Southern Sweden's training in Integrated Medicines Management as inspiration. Experienced clinical pharmacists in Norway have further developed and improved the program. The training program comprises i) a three-day in-class and skill training course in a simulation lab ii) eight days in a hospital ward performing medication reconciliations and - reviews under individual supervision from a clinical supervisor, i.e. an experienced clinical pharmacist, and iii) independently conducted medication reconciliations and –reviews presented to the supervisor for feedback and reflection.

**Results:**

Of 46 participants in the last four years, 39 (85 %) completed a questionnaire. Participants scored their overall satisfaction with a median of 6 (range 4–6) on a scale from 1 to 6. Participants highlighted the way the course is organized and facilitated by skilled lecturers, active learning with feedback, peer learning, and work relevance as positive factors.

**Conclusion:**

A comprehensive work-integrated training program with various and active learning methods that can be used as a template in settings where pharmacists graduate at novice level in medication reconciliation and -reviews has been developed. The program is appreciated by participants, seems to improve their professional confidence, and could ensure standardised high quality clinical pharmacy services.

## Highlights

• A comprehensive training program for clinical pharmacists has been developed.
• The program is work-integrated and comprises various and active learning methods.
• Suitable for pharmacists at novice level in medication reconciliation and -reviews.
• The training program seems to improve the pharmacistsʼ professional confidence.
• The program could ensure standardised high-quality clinical pharmacy services.

## 1 Introduction

To reduce medication-related harm and enhance patient safety, there is a growing recognition of the importance of medication reconciliation and reviews in clinical practice ^[^1, 2., 3^]^. In recent years, clinical pharmacy services have expanded rapidly in Norwegian hospitals, and in 2012 Integrated medicines management (IMM) was chosen as the working model of all clinical pharmacists conducting patient-oriented tasks in Norwegian hospitals ^[^^4^^]^. This systematic approach aims to tailor and optimize drug therapy for hospitalised patients, encompassing medication reconciliation upon admission, medication reviews during the hospital stay, and medication reconciliation along with patient counselling at discharge ^[^5., 6., 7.^]^. However, despite the acknowledgment of the importance of these tasks, patient-centered activities such as medication reconciliation and reviews are not sufficiently emphasized in the curricula of pharmacy at Norwegian universities, leading graduated pharmacists to be at the novice level. This gap highlighted the need for a postgraduate training program to enhance these core competencies for hospital-based clinical pharmacists.

The Hospital Pharmacies Enterprise South Eastern Norway comprises 20 hospital pharmacies and is located in the largest of four regional health regions in Norway that provide specialist healthcare services to 3.1 million people ^[^^8^^]^. In 2012, with inspiration from the IMM training in Southern Sweden ^[^^9^^]^, pharmacists from the Hospital Pharmacies Enterprise South Eastern Norway developed an in-house comprehensive training program for clinical pharmacists. Experienced clinical pharmacists took the role of clinical supervisors throughout the regional Norwegian program. Over the years, the training program has been continuously developed and improved. In the latter years, masterʼs students in pharmacy affiliated with the Research Group for Clinical Pharmacy at the University of Oslo, have attended the course together with postgraduate pharmacists. We have limited the scope of this paper to recent year's evaluations, that is 2021–2024. Currently, more than 120 participants have completed the program. The aim of this paper is to describe the program design and evaluation of the last four years (2021–2024).

## 2 Methods

### 2.1 Description of the training program

The aim of the training program is to standardise clinical pharmacy services and strengthen clinical pharmacists' theoretical knowledge, practical skills, and confidence in patient-centered tasks. The training program is included in the Hospital Pharmacies Enterprise South-Eastern Norway's competency development plan for clinical pharmacists. Completion of the program is required for clinical pharmacists working in a patient-oriented role. It is the head of the clinical pharmacy department in each hospital pharmacy that decides which of their clinical pharmacists should participate. Besides the training program described in the current paper, the competency development plan for clinical pharmacists also includes courses on subject matter offered from universities, e.g. on on clinical biochemistry in relation to medication use and pharmacological variability and individualized therapy.

In the training program a work-integrated learning (WIL) approach is used by integrating theory with meaningful practice in a safe learning environment ^[^^10^^]^. The program starts with a three-day in-class and skill training course in a simulation lab with 8–10 participants in each course. This is followed by eight days in a hospital ward performing medication reconciliations and –reviews under individual supervision and ends when a sufficient amount of independently conducted medication reconciliations and –reviews of satisfactory quality have been presented to the supervisor. A detailed description of the training program is shown in Table 1. Originally, the three-day in-class and skill training course was arranged as a course with physical attendance. The course in 2021 was however conducted fully digital due to the COVID-19 pandemic. In the following years, after experiencing that the third course day worked well in the digital format, the course was conducted as two days with physical attendance followed by a third digital day.

**Table 1:** Overview of the training program for clinical pharmacists conducting patient-oriented tasks in hospitals in South Eastern Norway.

Part of training program	Learning environment	Learning activities	Content
1) Three-day in-class and skill training course	Classroom, simulation lab, digital meeting	Lectures, skill training in simulation lab, feedback and reflection, video recording, small group case training	Introduction to IMM, theoretical and practical approach to medication reconciliation and -reviews, prioritization and communication of medication discrepancies and drug-related problems, hygiene in the hospital setting, pharmacist notes in the medical record, the discharge process and the transfer of information to the patient and the next care level.
2) Around eight days of clinical activities involving hospitalised patients under individual clinical supervision from experienced clinical pharmacists	Hospital ward at the supervisor's workplace	Course participant observing experienced clinical pharmacist (supervisor), course participant being observed by supervisor, feedback and reflection	First observe the supervisor conduct one medication reconciliation and -review, then self-conduct 10–20 medication reconciliations and 7–15 medicines reviews under supervision. This includes revealing medication discrepancies and drug-related problems, discussing them with ward physicians, and writing pharmacist notes in the patients' medical records.
3) Clinical work conducted independently at the course participant's workplace and presented to an experienced clinical pharmacist for feedback	Hospital ward at the course participant's workplace	Individual work, course participant presenting to supervisor, feedback, and reflection	Independently conduct a minimum of 10–20 medication reconciliations and a minimum of 5 medicines reviews and present the work to a supervisor retrospectively. The first presentation should be performed after 1–2 medication reconciliations and/or medicines reviews have been conducted, so feedback can be given before further independent work is conducted.

The number of medication reconciliations and –reviews that should be conducted is given as ranges allowing supervisors the flexibility to determine when participants demonstrate satisfactory skills in these tasks. This assessment is based on objective quality assessment forms. IMM = integrated medicines management.

#### 2.1.1 Skill training

**Fig. 1:**
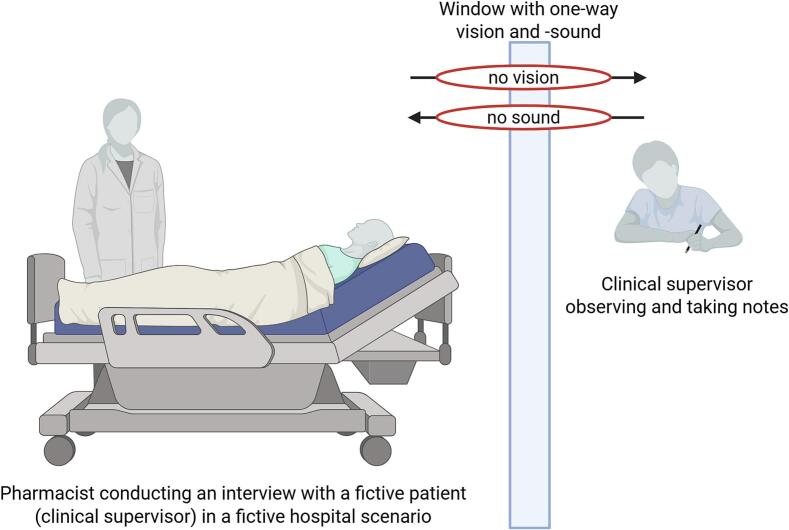
Skill training in the simulation lab in a fictive scenario. Created in BioRender. Lea, M. (2025) https://BioRender.com/ppjgkz4.

Skill training in medication reconciliation is conducted in a simulation lab, eCollaboration Laboratory (eColab) at the University of Oslo as illustrated in Fig. 1 ^[^^11^^]^. Each participant interviews a fictive patient, that is a clinical supervisor, in a fictive hospital scenario. Another clinical supervisor is observing and taking notes. Directly after the training session, the participant receives feedback one-to-one, and the participant and the supervisor reflect together. The interview is also video recorded, and each participant gets the opportunity to watch the recording afterward.

Furthermore, the participants practice medication reconciliations on two other fictive patient cases where the primary information sources are other than patient interview, e.g. the Norwegian Summary Care Record ^[^^12^^]^, the home nurse, nursing home staff, and next of kin. Skill training in medicines review is carried out in groups of 2–4 participants. They follow the standardised procedure for advanced medicines review ^[^^13^^]^ to reveal drug-related problems and prioritise them on the patient cases they already have practiced medication reconciliation on in the simulation lab.

Both the skill training session in medication reconciliations and the skill training session in medicines reviews end with a plenum session facilitated by the clinical supervisors in which the participants discuss each patient case. Here, short video clips from the participants' fictive patient interviews are shown after consent from the participant(s), to illustrate situations and/or communication skills that are relevant to reflect on, discuss, and learn from.

#### 2.1.2 Individual supervision and quality assessments

Individual supervision is initially provided to each participant for about eight days in a hospital ward at the supervisor's workplace, after which the participant will proceed with their individual work at their own workplace. This latter individual work is presented to a clinical supervisor for feedback and reflection, one-two patient cases at a time, in a period of up to 6 months. Each participant receives supervision from a minimum of two clinical supervisors, due to the usefulness to learn from several individual persons. The supervisors use quality assessment forms to ensure objective assessments of the participants clinical skills, see Supplementary material 1 and 2. The number of medication reconciliations and –reviews that should be conducted is given as ranges, which allows the supervisors to decide when the quality of participants skills is satisfactory, see Table 1. The quality assessment forms are presented to the participants during the in-class and skill training course, to give them clarity on what they are evaluated on.

#### 2.1.3 The clinical supervisors

Parallel to the training program a comprehensive follow-up of the supervisors has been conducted, in the latter years with a dedicated mentor (author ML). She has been available for discussions and advice, and organized meetings with relevant content for the supervisors to increase the supervisor's competence in the group. These meetings have been arranged in connection with each in-class and skill training course to prepare and align the supervisors for the upcoming supervision sessions.

The supervisors have been a relatively stable group of around ten experienced clinical pharmacists located in various hospital pharmacies in the region. None had much experience with clinical supervision from earlier. To build supervisor competence, a collaboration with an external educator with clinical supervision expertise (author NBØ) was started in 2014. She arranged and facilitated active learning such as reflection tasks, sharing experiences activities, and practical tasks in several of the meetings for the supervisors. She also gave lectures on the theory of supervision, adult learning, and feedback ^[^^14^^]^. Based on these lectures, a written guide for clinical supervision has been developed which contains a brief theory of supervision, presents two clinical supervision models, and advises on the supervisor role. In addition, the guide contains a checklist for both practical issues, e.g., to sign a non-disclosure agreement and to inform colleagues and relevant wards that supervision will take place, as well as focusing on the supervision itself.

#### 2.1.4 Costs

The in-house training program is offered free of charge to the participating pharmacists. As a result, the estimated costs of the program equal the lost working hours for participants, lecturers, and clinical supervisors, as well as travel expenses for participants residing far from the course location.

### 2.2 Evaluation of the training program

A digital questionnaire with a combination of closed- and open-ended questions was answered by the participants after the three-day in-class and skill-lab course. The close-ended questions were designed as a Likert scale ranging from 1 “to a very small extent” to 6 “to a very large extent”. The open-ended questions invited the participants to respond to questions in their own words and to share details about their experiences to “capture the ‘why’ that complements quantitative results, helping to tell a more nuanced story with the data” ^[^^15^^]^.

All questions are shown in Table 2. The questions marked with grey color are considered the most important locally, and the least relevant for a broader audience. These questions are therefore not analysed in the current paper.

**Table 2:** The questions of the digital questionnaire that were distributed to all the participants.

	Questions	Option for answers
1	Overall, how satisfied are you with the course?	Likert scale
2	To what extent did the course match your expectations?	Likert scale
3	To what extent are you satisfied with the information you received before the course?	Likert scale
4	To what extent has the course made you more confident in meeting with the clinic when performing patient-related tasks like medication reconciliation and -review?	Likert scale
5	To what extent did you benefit from the skill training in medication reconciliation in the simulation lab: a. To conduct the patient interview? b. To receive one-to-one feedback from a clinical supervisor? c. To watch the video recording of your interview performance afterward? 1 d. The plenary discussion after the skill training? 1	a-d: Likert scale
6	To what extent do you think the course instructors: a. Were professionally competent? b. Conveyed the course material in a good way?	a-b: Likert scale
7	What is your opinion on the time spent on: a. Lectures with theory b. Working with cases c. Plenary discussions of cases	a-c: Likert scale
8	To what extent did you experience Skype/Teams as a suitable learning environment for the third course day? 2	Likert scale
9	What was the best part of the course?	Open-ended
10	Were there lacking topics in the course?	Open-ended
11	Where do you think the course has potential for improvement?	Open-ended
12	Do you have any other comments about the course?	Open-ended

The Likert scale ranged from 1 “to a very small extent” to 6 “to a very large extent”. The questions marked with grey color are not analysed in the current paper.

1 The question was not a part of the questionnaire in 2021.

2 In August 2021 the question ended with “…for the course?” since the course was arranged as a fully digital course due to the COVID-19-pandemic.

The answers on the close-ended questions 1, 4, 5, and 8 were analysed in IBM SPSS Statistics 29.0.1.0. The answers to the open-ended questions 9–12 were analysed using thematic analysis by Braun and Clarkes ^[^^16^^]^ and their stepwise guide to identify themes within the data. First, we familiarized ourselves with the data. Thereafter we generated initial codes and searched for themes, and then we finally defined and named the themes. Two of the authors (ML and NBØ) were mainly responsible for the analysis process. All authors participated in the final discussion of the analysis that took place in an online meeting.

#### 2.2.1 Ethics

The study was evaluated by the Data Protection Officer at the Hospital Pharmacies Enterprise, South Eastern Norway, and recommendations were provided based on anonymised data.

## 3 Results

Of the 46 course participants attending the five three-day in-class courses arranged in the last four years, 39 (85 %) completed the digital questionnaire. Table 3 shows an overview of the number of participants in each course over the last four years and the proportion of responses on the evaluation.

**Table 3:** Overview of the number of participants on each three-day in-class course, and the proportion of participants completing the evaluation, that is a digital questionnaire.

Month and year the course was arranged	Number of participants attending the course	Proportion (number) of participants completing the evaluation
August 2021^1^	14	86 % (12)
August 2022	9	56 % (5)
November 2022	7	100 % (7)
August 2023	11	100 % (11)
April 2024	5	80 % (4)

1 Fully digital course due to the COVID-19 pandemic.

The participants scored their overall satisfaction with the course with a median of 6 (range 4–6). None of the 39 respondents answered 1 to any of the questions. An overview of participant responses to the individual close-ended questions is shown in Table 4.

**Table 4:** Responses to the individual close-ended questions in the digital questionnaire on the 6-points Likert scale ranging from 1 “to a very small extent” to 6 “to a very large extent”.

Question	Likert scale					
	1	2	3	4	5	6
Overall, how satisfied are you with the course?	0 (0)	0 (0)	0 (0)	1 (3)	16 (41)	**22 (56)**
To what extent has the course made you more confident in meeting with the clinic when performing patient-related tasks like medication reconciliation and -review?	0 (0)	0 (0)	2 (5)	4 (10)	**16 (41)**	17 (44)
To what extent did you benefit from the skill training in medication reconciliation in the simulation lab:						
a. To conduct the patient interview?	0 (0)	0 (0)	0 (0)	2 (5)	5 (13)	**32 (82)**
b. To receive one-to-one feedback from a clinical supervisor?	0 (0)	0 (0)	0 (0)	1 (3)	5 (13)	**33 (85)**
c. To watch the video recording of your interview performance afterward? 1	0 (0)	0 (0)	2 (7)	6 (22)	3 (11)	**16 (59)**
d. The plenary discussion after the skill training?^1^	0 (0)	0 (0)	0 (0)	1 (4)	8 (30)	**18 (67)**
To what extent did you experience Skype/Teams as a suitable learning environment for the third course day?^2^						
• All 39 responses	0 (0)	2 (5)	4 (10)	5 (13)	**11 (28)**	17 (44)
• 2021 (fully digital), 12 responses	0 (0)	2 (17)	2 (17)	**4 (33)**	2 (17)	2 (17)
• 2022–24 (third day digital), 27 responses	0 (0)	0 (0)	2 (7)	1 (4)	9 (33)	**15 (56)**
						

The answers to each response are presented in numbers (%). The median is indicated in bold.

1 The question was not a part of the questionnaire in 2021, hence 27 responses.

2 In August 2021 the question ended with “…for the course?” since the course was arranged as fully digital course due to the COVID-19-pandemic.

The results from the open-ended questions in the questionnaire revealed both positive and negative factors for learning, as well as suggestions for improvement. Table 5 shows all the main themes with sub-themes and examples.

**Table 5:** Themes and sub-themes representing what the participants reported in the open-ended questions.

Main theme	Sub-theme	Examples from questionnaire responses
Positive factors	Organization and facilitation	• *Structured course program, good and thorough presentation, and very skilled lecturers.* • *The theory part, group tasks, and feedback in plenary sessions [were the best part of the course].* • *Very useful to get an overview of the theory before conducting practical tasks.*
	Active learning and feedback	• *The best part of the course was completing a medication reconciliation, receiving feedback, as well as watching video recordings afterward to be able to learn from the mistakes.* • *Practicing patient cases was extremely useful and receiving feedback afterwards.* • *A little out of my comfort zone with the simulation at EcoLab, but especially useful with feedback right afterward and being able to see myself afterwards.*
	Peer learning	• *Meeting other colleagues, but it was also interesting that there were [undergraduate] students [that attended the course] and those who were new to working life as they brought other perspectives and might have undergone other subjects during their education compared to oneself. That was extremely helpful.* • *Working with patient cases in small groups (2–4 course participants) made it easy to collaborate and discuss things without taking too long.* • *The group tasks were especially useful, as you got to discuss with others and hear their views.* • *Nice that we were in Oslo [in-person session] for the first two days. I'm not sure if the last day on Teams would have worked so well if we hadn't met in real-life before and get to know each other better.*
	Work relevance	• *It was a particularly useful course in practicing medication reconciliation.* • *It was great to practice a patient interview. I believe that this will improve the quality of my first conversation with a real patient and my entrance to the hospital setting.*
Negative factors	1 Technical aspects	• *Technical difficulties in digital sessions.*
	1 Digital learning environment	• *Less sharing and peer learning in digital sessions.*
Suggestions for improvement	Practical work-relevant tasks	• *Increased amount of fictive patient cases with various challenges.* • *More documentation of clinical tasks in the medical record.* • *Examples and feedback on the practical use of the medication reconciliation form.* • *Spending increased time on stepwise practical use of the medication review form.*
	Training in groups	• *Increased amount of small group sessions and more time to work in groups.*
	Guidelines	• *More on treatment guidelines and how to find them.*

1 Only reported related to the fully digital course in 2021.

## 4 Discussion

The current paper presents a comprehensive work-integrated training program designed to address the core competencies of clinical pharmacists, specifically focusing on medication reconciliation and reviews. The training program has been developed in-house in the Hospital Pharmacies Enterprise South Eastern Norway to bridge the gap between the skills pharmacists need to deliver the well-documented IMM model to ensure medication safety ^[^^17^^]^ and the competencies that are inadequately addressed in current university curricula. Evaluations of the training program conducted over the last four years revealed median scores of 5 or 6 on all questions on a Likert scale ranging from 1 to 6, indicating a high level of participant satisfaction. Overall satisfaction with the program received a median score of 6, with no respondents rating their experience below a score of 4. The training program has the potential to serve as a model for settings where newly graduated pharmacists possess limited proficiency in clinical pharmaceutical skills such as medication reconciliation and -reviews.

Over the past decade, a Finnish hospital pharmacy has also developed an in-house training program ^[^^18^^]^. While our program focuses heavily on volume training on methodology in practical skills under close supervision, the Finnish program appears to place a greater emphasis on expert lectures such as interpreting laboratory results, medication use related to renal and hepatic insufficiency, and literature related to one's own area of specialty. In Norway such specific academic courses are offered by educational institutions, e.g. on clinical biochemistry in relation to medication use, and pharmacological variability and individualized therapy. While there undoubtedly are discussions on subject matter during the volume training in medication reviews methodology, the course instructors emphasize to participants that our primary focus is developing skills in *the application of their knowledge on subject matter*. This difference between the countries is reflected by the significantly higher number of medication reconciliations and reviews that participants must complete during our program, totaling 20–40 medicines reconciliations and 12–20 medication reviews, versus the numbers in Finland are 4 medication reconciliations and 9 medication reviews ^[^^18^^]^. The content of the undergraduate curriculum and the postgraduate courses offered from e higher education institutions in each specific setting will be crucial in determining the best way to design in-house training programs for clinical pharmacists. Other features that seem unique to our training program compared to the Finnish one is the use of a simulation lab for skill training and the parallel training of clinical supervisor group.

An aim of our training program is to strengthen clinical pharmacists' theoretical knowledge, practical skills, and confidence in patient-centered tasks. Through the open-ended questions, the participants highlighted the way the course is organized and facilitated by skilled lecturers, active learning with feedback, peer learning, and work relevance as positive factors. Furthermore, almost 9 out of 10 participants rated their self-confidence dividend of the course in the meeting with the clinic as 5 or 6 on a Likert scale ranging from 1 to 6. This is an important finding since professional confidence seems to be important for optimal patient safety ^[^^19^^]^. Altogether, the evaluation indicates good alignment between the intended learning outcomes, the learning activities, and the formative feedback and -reflection sessions with a supervisor ^[^^20^^]^.

The skill training sessions conducted as simulation with feedback afterward and learning from peers were emphasized as especially valid for the participants' learning. Simulation as a learning method creates a safe environment for learning skills and has been shown to improve healthcare self-efficacy, patient outcomes, and patient safety in various settings in healthcare ^[^21., 22, 23., 24^]^. The impact of peer learning interaction also seems to have contributed to the overall satisfaction of the course according to the participantsʼ answers. This way of facilitating learning is anchored in constructivist learning theory emphasising dialog, collaboration, and development of shared knowledge and meaning of content ^[^^25^^]^. Furthermore, the active learning approach utilised in our training program is related to the pedagogical principles of continuous professional development (CPD), that is learning-centered and self-directed, ongoing, and systematic with a learning outcomes approach and linking learning into practical context ^[^^26^^]^. CPD has been described to be essential for healthcare professionals to remain current with best practices, and helps to address gaps in their knowledge and skills ^[^^27^^]^. Our training program is tailored for clinical pharmacists in the hospital setting. However, the program could stimulate advancements in healthcare professionals' continuous professional development related to other settings or other clinical tasks.

To facilitate a safe learning environment and plenary sharing and discussions it is important to facilitate networking and learning from peers, which have been recognized as a preferred learning method of pharmacists themselves ^[^^28^^]^. Our training program is therefore limited to 8–10 participants. Furthermore, the opportunity to practice what they have learned at the supervisor's workplace after the three-day in-class course is important for further learning and developing their competencies. At last, the independent work integrated at the participantﹸs workplace with close follow-up by the supervisor is assumed to be particularly useful since this follow-up extends over a certain period, that is weeks to months, contains both discussions around real patient cases and other clinical work-related issues, making it a form of mentoring. Previous studies have pinpointed the importance of postgraduate training for pharmacists both to build their confidence and result in success in practice, and mentoring seems to be a promising approach to achieve this ^[^^28^^,^^29^^]^. Importantly, with a work-integrated training program across physical locations, the organization ensures that the competence needed for high-quality services is spread throughout all hospital pharmacies in the region ^[^^3^^]^.

In 2021, the three-day in-class course was conducted fully digital due to the COVID-19 pandemic. The participants in this specific course mentioned technical aspects and the digital learning environment as negative factors for learning. An important learning experience was, however, that the third course day worked well in the digital format, leading to arranging the following courses as two days with physical attendance and the third day digital, with the positive effect of increasing flexibility and reducing costs. The fact that negative factors were not mentioned in the following courses implies that the third digital course day worked well when the participants had met two days in person before and got to know each other and also underlines the participants' general high satisfaction with the course.

On the improvement potential, participants seemed to generally want more, both more practical work-relevant tasks, more training in groups, and additional knowledge on treatment guidelines and how to use them. While our training program does not cover the latter, as it primarily focuses on the methodologies for clinical pharmacists, current improvements and adjustments consist of increasing the amount of practical work-relevant tasks and training in groups moving towards blended learning. The blended learning approach includes pre-learning activities, such as video lectures and assigned readings, to prepare participants before attending the in-person course. Furthermore, skill training in the simulation lab that focuses on discussing medication discrepancies and drug-related problems with a fictive physician is currently being explored. Blended learning is increasingly recognized as the future of health professions education ^[^^30^^]^. By integrating this model, course facilitators could enhance face-to-face interactions, fostering social engagement and enabling a thorough assessment of the participants' practical skills during the in-person sessions ^[^^30^^]^. This is particularly important as participants have emphasized the value of simulation lab sessions and the reflections and discussions with peers for their learning outcomes. Looking ahead, the training program could also benefit from incorporating interprofessional training alongside physicians and nurses, both in the skill lab and hospital setting, to make the pharmacists fit for the collaborative practice required to meet the current increasingly complex needs in healthcare ^[^^31^^]^.

As many as 85 % of the clinical pharmacists who have attended the course in the last four years responded to the questionnaire, which gives a good picture of the participants' learning outcomes and their opinions about the course. However, a key limitation is that the evaluation relies on self-reported learning outcomes of participants which may not necessarily reflect actual improvements in clinical competence or changes in behavior. While the objective quality assessment forms used by the supervisors ensure that participants demonstrate satisfactory clinical skills, future evaluations could benefit from being more advanced. Specifically, they should focus on assessing behavior changes in participants, knowledge transfer, and the impact on clinical performance after attending the training program, in line with Kirkpatrick's Four-Level model ^[^^32^^]^, even if this is challenging. On patient outcomes, we have indirect data since all clinical pharmacists who have served as data collectors and conducted medication reconciliations and -reviews in recent studies in Norway ^[^^13^^,^^33^^]^, first completed the comprehensive training program that is described in the current paper. Pharmacists completing the training program have revealed medication discrepancies in nine out of ten patients at hospital admission ^[^^33^^]^. Furthermore, it seems reasonable to suggest that the comprehensive training program could have contributed to the 34 % increased overall survival seen in the intervention group receiving Integrated Medicines Management (IMM) in a Norwegian randomized controlled trial ^[^^13^^]^.

Three of the authors are or have been clinical supervisors and facilitated the training program for many years. This can be considered both a strength due to the deep insight, and a limitation due to the risk of subjective views.

## 5 Conclusion

A comprehensive work-integrated training program with various and active learning methods that can be used as a template in settings where pharmacists graduate at novice level in medication reconciliation and -reviews has been developed. The program is appreciated by participants, seems to improve their professional confidence, and could ensure standardised high quality clinical pharmacy services.

## CRediT authorship contribution statement

**Marianne Lea:** Writing – review & editing, Writing – original draft, Visualization, Methodology, Formal analysis, Conceptualization. **Elin Trapnes:** Writing – review & editing, Methodology, Formal analysis, Conceptualization. **Hanne Steen:** Writing – review & editing, Methodology, Formal analysis, Conceptualization. **Nina Bjerketveit Ødegaard:** Writing – review & editing, Methodology, Formal analysis, Conceptualization.

## Declaration of generative ai and ai-assisted technologies in the writing process

During the preparation of this work the author used GPT UiO in order to improve the readability and language of a couple of paragraphs in the manuscript. After using this tool, the authors reviewed and edited the content as needed and takes full responsibility for the content of the published article.

## Funding

This research did not receive any specific grant from funding agencies in the public, commercial, or not-for-profit sectors.

## Declaration of competing interest

The authors declare that they have no known competing financial interests or personal relationships that could have appeared to influence the work reported in this paper.

## Data Availability

All data are available in the paper.

## Appendix A Supplementary data

Quality assessment of medicines reconciliation (MedRec) - a checklist. Image 1

Quality assessment of medicines review (MedRev) – a checklist. Image 2
