# Supplementary material for: Design and evaluation of a comprehensive training program for hospital-based clinical pharmacists - various, active, and work-integrated learning
Source: Explor Res Clin Soc Pharm. 2025 Oct 27;20:100677. doi: 10.1016/j.rcsop.2025.100677 (PMC12666581; doi:10.1016/j.rcsop.2025.100677)
Supplement: Supplementary file 1 — Quality assessment of medicines reconciliation (MedRec) - a checklist. [file mmc1.pdf]

QUALITY ASSESSMENT OF MEDICINES RECONCILIATION (MedRec) – a checklist

Course participant:____________________

Clinical supervisor:_______________________

MedRec number:__________________

Date presented to clinical supervisor:_______________

The course participant should strive to achieve «YES» (or «NOT APPLICABLE») on all questions. In some cases «PARTIALLY» can at discretion be assessed as approved.

1. Is the procedure followed?

   YES ☐ NO ☐ PARTIALLY ☐

2. If observation: Is the communication with the information sources clear?

   YES ☐ NO ☐ PARTIALLY ☐ NOT APPLICABLE ☐

3. Is medication handling before hospital admission assessed? <u>(Must be fulfilled!)</u>

   YES ☐ NO ☐

4. Is the use of multidose drug dispencing assessed? (If the patient has multidose, is the medication list obtained?)

   YES ☐ NO ☐

5. Is the information source closest to the medication handling used? (If NO it must be justified why not.)

   YES ☐ NO ☐ PARTIALLY ☐

6. Is the patient used as an information source? (If NO it must be justified why not.)

   YES ☐ NO ☐ PARTIALLY ☐

7. Are all uncertainties on the patient's regular drug use clarified?

   YES ☐ NO ☐ PARTIALLY ☐ NOT APPLICABLE ☐

8. Are all necessary information sources obtained? (If NO it must be justified why not.)

   YES ☐ NO ☐ PARTIALLY ☐ NOT APPLICABLE ☐

9. Are all questions in the checklist asked?

   YES ☐ NO ☐ PARTIALLY ☐ NOT APPLICABLE ☐

10. Is the obtained information noted in the form according to the procedure?

    YES ☐ NO ☐ PARTIALLY ☐

11. Is the patient's actual use of medications on demand revealed?

    YES ☐ NO ☐ PARTIALLY ☐ NOT APPLICABLE ☐

12. Are all relevant medication discrepancies discussed with a physician? <u>(Must be fulfilled!)</u>

    YES ☐ NO ☐

13. If observation: When discussing relevant medication discrepancies with the physician, is the communication clear?

    YES ☐ NO ☐ PARTIALLY ☐ NOT APPLICABLE ☐

Approved to independent level: YES☐ NO ☐ Remaining work:___________________________

Deadline for remaining work:_______________________________________
