# Supplementary material for: Design and evaluation of a comprehensive training program for hospital-based clinical pharmacists - various, active, and work-integrated learning
Source: Explor Res Clin Soc Pharm. 2025 Oct 27;20:100677. doi: 10.1016/j.rcsop.2025.100677 (PMC12666581; doi:10.1016/j.rcsop.2025.100677)
Supplement: Supplementary file 2 — Quality assessment of medicines review (MedRev) – a checklist. [file mmc2.pdf]

QUALITY ASSESSMENT OF MEDICINES REVIEW (MedRev) – a checklist

Course participant:____________________

Clinical supervisor:_______________________

MedRev number:__________________

Date presented to clinical supervisor:_______________

The course participant should strive to achieve «YES» (or «NOT APPLICABLE») on all questions. In some cases «PARTIALLY» can at discretion be assessed as approved.

1. Is the field "Background information" filled according to the procedure?
   YES □ NO □ PARTIALLY □ NOT APPLICABLE □

2. Outdraws of the medical journal are relevant for the patiens't medication use (indication (for example symptoms), adverse effects, conditions that alter medication metabolism, - elimination, and - dosage), and are concise. Medication names are in red writing.
   YES□ NO □ PARTIALLY □ NOT APPLICABLE □

3. Laboratory tests and clinical measurements are documented correctly in the form?
   YES□ NO □ PARTIALLY □ NOT APPLICABLE □

4. Therapeutic drug monitoring (TDM) medications included in the procedure are assessed, including specific suggestions for action (e.g. when the monitoring sample is to be taken, which sample(s) should be taken, frequency, or a justification for not monitoring) and/ or results are available/documented? YES□ NO □ PARTIALLY □ NOT APPLICABLE □

5. Potentially inappropriate medication use in older adults (e.g. according to START/STOPP) is assessed and specific suggestions for action/results are documented?
   YES□ NO □ PARTIALLY □ NOT APPLICABLE □

6. Medication handling issues are documented and specific suggestions for action/results are documented? YES□ NO □ PARTIALLY □ NOT APPLICABLE □

7. Clinically relevant drug-drug interactions are documented and specific suggestions for action/results are documented? YES□ NO □ PARTIALLY □ NOT APPLICABLE □

8. Medication dose adjustment because of reduced organ function and contraindication are assessed and specific suggestions for action/results are documented?
   YES□ NO □ PARTIALLY □ NOT APPLICABLE □

9. All medications are assessed for therapeutic indication, and for medications without a clear indication, there are specific suggestions for action/results documented?
   YES□ NO □ PARTIALLY □ NOT APPLICABLE □

10. All short-term use of medications (course medication) are documented with starting date and recommended length of treatment?
    YES□ NO □ PARTIALLY □ NOT APPLICABLE □

11. All former/new indications, new symptoms, and laboratory results outside the reference range are assessed according to optimal medication treatment?
YES□ NO □ PARTIALLY □ NOT APPLICABLE □

12. Are the medication treatment for every therapeutic indication *assessed* and *found reasonable* according to existing international, national, or local treatment guidelines?
YES□ NO □ PARTIALLY □ NOT APPLICABLE □

13. Symptoms or laboratory results outside the reference range that can be adverse effects are assessed according to the patient's medication use?
YES□ NO □ PARTIALLY □ NOT APPLICABLE □

14. Drug-related problem (DRP) documented under the category «other» does not belong under any other category?
YES□ NO □ PARTIALLY □ NOT APPLICABLE □

15. Medication discrepancies identified during medication reconciliation are documented correctly, including suggestions for action? YES□ NO □ PARTIALLY □ NOT APPLICABLE □

16. Are all DRP categories documented with the date and the signature of the person who revealed the DRP? YES□ NO □ PARTIALLY □ NOT APPLICABLE □

17. Are all DRPs that have been presented to relevant healthcare professionals documented with the date and the pharmacist's signature, and the DRPs not presented not signed?
YES□ NO □ PARTIALLY □ NOT APPLICABLE □

18. Are the suggestions for action to the DRPs specific (who, what, which dosages, several possible solutions when applicable)? YES□ NO □ PARTIALLY □ NOT APPLICABLE □

19. Are the initials of the person the DRP is presented to documented?
YES□ NO □ PARTIALLY □ NOT APPLICABLE □

20. When more than one DRP is revealed in a DRP category; is it clear which suggestion belongs to which DRP? YES□ NO □ PARTIALLY □ NOT APPLICABLE □

21. Is the box «Medicines review conducted" signed according to the procedure?
YES□ NO □ PARTIALLY □ NOT APPLICABLE □

22. Are relevant DRPs presented to a physician? If not, is it explained why?
YES□ NO □ PARTIALLY □ NOT APPLICABLE □

The course participant and the supervisor agree on the following need/priorities for further training:

Approved to independent level: YES□ NO □ Remaining work:___________________________

Deadline for remaining work:_______________________________________
